# Cardiovascular magnetic resonance by non contrast T1-mapping allows assessment of severity of injury in acute myocardial infarction

**DOI:** 10.1186/1532-429X-14-15

**Published:** 2012-02-06

**Authors:** Erica Dall'Armellina, Stefan K Piechnik, Vanessa M Ferreira, Quang Le Si, Matthew D Robson, Jane M Francis, Florim Cuculi, Rajesh K Kharbanda, Adrian P Banning, Robin P Choudhury, Theodoros D Karamitsos, Stefan Neubauer

**Affiliations:** 1 Oxford Centre for Clinical Magnetic Resonance Research, Department of Cardiovascular Medicine, University of Oxford, John Radcliffe Hospital, Oxford, OX3 9DU, UK; 2 The Wellcome Trust Centre for Human Genetics, Roosevelt Drive, Oxford, OX3 7BN, UK; 3 Department of Cardiology, John Radcliffe Hospital, Oxford, OX3 9DU, UK; 4 Department of Cardiovascular Medicine, University of Oxford, John Radcliffe Hospital, Oxford, OX3 9DU, UK

## Abstract

**Background:**

Current cardiovascular magnetic resonance (CMR) methods, such as late gadolinium enhancement (LGE) and oedema imaging (T2W) used to depict myocardial ischemia, have limitations. Novel quantitative T1-mapping techniques have the potential to further characterize the components of ischemic injury. In patients with myocardial infarction (MI) we sought to investigate whether state-of the art pre-contrast T1-mapping (1) detects acute myocardial injury, (2) allows for quantification of the severity of damage when compared to standard techniques such as LGE and T2W, and (3) has the ability to predict long term functional recovery.

**Methods:**

3T CMR including T2W, T1-mapping and LGE was performed in 41 patients [of these, 78% were ST elevation MI (STEMI)] with acute MI at 12-48 hour after chest pain onset and at 6 months (6M). Patients with STEMI underwent primary PCI prior to CMR. Assessment of acute regional wall motion abnormalities, acute segmental damaged fraction by T2W and LGE and mean segmental T1 values was performed on matching short axis slices. LGE and improvement in regional wall motion at 6M were also obtained.

**Results:**

We found that the variability of T1 measurements was significantly lower compared to T2W and that, while the diagnostic performance of acute T1-mapping for detecting myocardial injury was at least as good as that of T2W-CMR in STEMI patients, it was superior to T2W imaging in NSTEMI. There was a significant relationship between the segmental damaged fraction assessed by either by LGE or T2W, and mean segmental T1 values (P < 0.01). The index of salvaged myocardium derived by acute T1-mapping and 6M LGE was not different to the one derived from T2W (P = 0.88). Furthermore, the likelihood of improvement of segmental function at 6M decreased progressively as acute T1 values increased (P < 0.0004).

**Conclusions:**

In acute MI, pre-contrast T1-mapping allows assessment of the extent of myocardial damage. T1-mapping might become an important complementary technique to LGE and T2W for identification of reversible myocardial injury and prediction of functional recovery in acute MI.

## Background

Cardiovascular magnetic resonance (CMR) with late gadolinium enhancement (LGE) is the current gold standard for assessing myocardial scar in chronic coronary artery disease [1,2], while T2-weighted (T2W) CMR is the accepted method for detecting oedema in acute ischemic injury [3]. Myocardial oedema can be detected by T2W-CMR as early as 30 minutes after the onset of ischemia, and in the absence of LGE, is thought to represent reversible myocardial injury [3,4]. LGE and T2W-CMR are used as complementary techniques, to provide a comprehensive assessment of irreversible and reversible injury in acute myocardial infarction (MI) and to derive important prognostic indices such as salvageable area-at-risk and scar burden [5].

The clinical significance of LGE as a precise measure of myocardial irreversible injury has recently been challenged. Firstly, in the early hours post ischemia, dynamic changes of LGE were shown within the ischemic myocardium, together with a significant regression of LGE over time and full functional recovery; these findings would suggest that acute LGE does not always represent scar [6-9]. Secondly, it was shown that early gadolinium enhancement can detect salvageable myocardium [10], indicating that the kinetics of gadolinium may play an important role in assessing area at risk in relation to the imaging time. It is also well known that the area of injury detected by T2W-CMR encompasses both irreversibly injured myocardium and salvageable myocardium at-risk [3], and that it may evolve in the early days post acute MI with significant variation in the resulting calculated volume of salvaged myocardium [6]. Furthermore, the concept that T2W positive imaging findings represent the area at risk has also been challenged recently [11] due to the pitfalls of the technique, although newer T2prep bright blood sequence may overcome most of the limitations inherent to fast spin echo based techniques [12,13]. Finally, there are important issues with the threshold-based methods widely used to post-process oedema and LGE images. Firstly, there is considerable scope for error depending on the threshold used, and secondly it remains unclear whether or not an arbitrary signal intensity threshold realistically reflects the tissue changes occurring in the myocardium [14,15].

Given these challenges and the heterogeneous composition of the acutely injured myocardium, a method that can objectively and directly quantify the extent and severity of acute ischemic injury would be highly desirable. Pre-contrast T1-mapping may be such a tool: compared to T2W and LGE, the main advantage of T1-mapping is that it provides measurements of absolute T1 relaxation times for each pixel, with high resolution [16,17]. Although T1-mapping is not exempt from the need for thresholding when sizing myocardial injury, a threshold-based post-processing method requiring reference ROIs (whether in remote myocardium or skeletal muscle) is not needed. This makes quantitative techniques such as T1-mapping less prone to subjectivity and error. In experimental models of acute MI, elevations of absolute T1values have been demonstrated in areas of increased water content exceeding the area of infarction by histological assessment [18,19]. Other studies suggested that further increases in T1 values occur with prolonged ischemia due to larger concentrations of other water constituents [19]. These studies indicate the potential of T1-mapping to detect areas of myocardial ischemia in humans. Indeed, early proof of principle studies have demonstrated increased T1 values in areas of positive LGE following acute MI [20,21]. However, previous T1-mapping techniques were of limited use in clinical practice, particularly in acutely ill patients, due to long breath-holds and inability to work well with increased heart rates. We have recently developed and validated a new pre-contrast T1-mapping sequence (Shortened Modified Look-Looker Inversion recovery (ShMOLLI) [17], which is highly suitable for examining patients with acute MI, because it not only allows for significantly shorter breath-hold time, but also provides a more accurate estimate of long T1values at higher heart rates [17]. Furthermore the T1 values can be directly assessed during the CMR scan without the need for post-processing.

Using this powerful new technique, the aims of this study were to investigate whether in patients with acute myocardial infarction, pre-contrast T1-mapping (1) detects acute myocardial injury, (2) is able to quantify the severity of myocardial damage when compared to standard techniques such as LGE andT2W, and (3) has the ability to predict long term functional recovery.

## Methods

### Patient population

This prospective study was undertaken in a single tertiary center. The local ethics committee approved the study protocol, and all patients gave written informed consent. Patients with first occurrence of acute MI were included. Myocardial infarction was defined according to a history of symptoms consistent with acute myocardial ischemia, with or without ST-segments elevation on the ECG associated with a rise in troponin I concentration [22,23]. Patients with previous MI, previous revascularization procedure (coronary artery bypass grafts [CABG] or percutaneous coronary intervention [PCI]), severe heart valve disease, known cardiomyopathy or hemodynamic instability lasting longer than 12 hours following revascularization were not enrolled. Further exclusion criteria were contraindications to CMR, including implanted pacemakers, defibrillators, or other metallic implant.

Acute clinical management was at the discretion of the responsible physician, with the intention to reflect contemporary practice and guidelines (including use of aspiration catheters; glycoprotein IIb/IIIa receptor inhibitors and high-dose clopidogrel loading).

### CMR

Patients underwent CMR 12 - 48 hours after the onset of chest pain and at 6 months (6M). STEMI patients underwent primary PCI prior to CMR in acute setting. CMR examinations were performed on a 3 Tesla MR scanner (TIM-Trio, Siemens Healthcare, Erlangen, Germany) using aspine and a phased array 6-channel flexible surface coils. Short axis cine images covering the length of the ventricle were acquired as previously reported [6]. Slices matching the cine positions were acquired using T2W, T1-mapping and LGE imaging (Additional file 1) with full coverage of the left ventricle. Steady-state free precession (SSFP) cine images were acquired using retrospective gating (TE/TR = 1.4/3.2 msec; flip angle = 50°; pixel size: 1.6 × 1.6 mm). Two to three-fold accelerated parallel imaging (GRAPPA) was used to shorten the breath-hold. Oedema imaging (T2W) was performed using a T2-prep-SSFP single shot sequence with surface coil correction [12] (TE/TR = 1/4.1 msec; effective TE = 60 msec; flip angle = 90°;pixel size: 2.1 × 1.6 mm). ShMOLLI T1 maps were generated from 5-7 SSFP images with variable inversion preparation time as described previously [17]. Typical acquisition parameters were: TE/TR = 1.07/2.14 msec, flip angle = 35°, FOV = 340 × 255 mm, matrix size = 192 × 144, 107 phase encoding steps, actual experimental pixel size = 1.8 × 1.8 mm, interpolated reconstructed pixel size = 0.9 × 0.9 mm, GRAPPA = 2, 24 reference lines, cardiac delay time TD = 500 ms and 206 ms acquisition time for single image, phase partial Fourier 6/8. If necessary, shimming and center frequency adjustments were performed before T2W-imaging and T1-mapping to generate images free from off-resonance artefacts. LGE-CMR was performed with a T1-weighted segmented inversion-recovery gradient echo-phase sensitive-inversion recovery (GRE_PSIR) sequence [24] (TE/TR = 2.5 msec/5 msec, voxel size 1.8 × 1.4 mm, flip angle 20°). Images were collected 10 to 15 minutes after the administration of 0.1 mmol/kg contrast agent (Gadodiamide, Omniscan™, GE Healthcare, Amersham, UK). The inversion time was meticulously adjusted for optimal nulling of remote normal myocardium.

All patients were in sinus rhythm during the CMR scan. The scan time needed to acquire a short axis stack with full coverage of the ventricle was about 2 min using ShMOLLI versus 4 min using T2W.

### Image analysis

Quantification of LV volumes and ejection fraction (EF) was performed as previously described using Argus software (Version 2002B, Siemens Medical Solutions)[25].

All matching short axis images covering the LV and acquired using each of the techniques (T2W, LGE and T1 maps) were manually contoured using an in-house software MC-ROI (IDL v.6.1, http://www.ittvis.com) to outline the endo- and epicardium. For the comparisons among acute T1-mapping, T2W and LGE, we excluded apical slices due to partial volume effects slices including the LVOT and slices with off-resonance artifacts. Each slice was segmented in a 6 equiangular segment model with the RV-LV junction as reference point. For analyses purposes, the slices acquired with different techniques were all matched for slice position and radially aligned to the positions of the papillary muscles and the LV-RV junction.

A total of 129 slices acquired acutely were suitable for analyses (on average 3.1 ± 0.9 slices per patient with a range of 1 to 5). Out of the resulting 774 segments, a total of 100 segments (13%) were excluded from analysis due to: (i) SSFP off resonance artifacts on T1-mapping or T2W images (70 segments) and (ii) presence of outflow tract (30 segments) on LGE or cine images.

The signal intensity threshold indicating oedema/LGE was set at 2 standard deviations (SD) above the mean intensity of reference ROI placed in remote unaffected myocardium as previously described [26,27]. Although different thresholds have previously been applied [5], there is no standardization and agreement: we used 2 SD for both techniques to avoid introducing systematic errors by applying different thresholds for T2W versus LGE (i.e. 2 SD versus 5 SD) and overestimating the area of salvaged myocardium [14]. Per segment, we derived the average T1 values and the signal intensity normalized to the signal intensity of the remote myocardium both for LGE and T2W. The coefficient of variation (CV = standard deviation/mean) of T2W signal intensities and T1 values were assessed in remote normal myocardium to detect the variability of measurements.

The injured fractions by LGE and oedema both segmental (volume of injured myocardium within a segment/volume of the segment) and global (volume of injured myocardium within the LV/LV mass) were assessed. Salvaged myocardial index was derived from the volume of acute oedema and the final infarct size (LV volume of LGE at 6M). Microvascular obstruction (MO)/haemorrhage was identified as the low intensity core if present on all analysed CMR sequences and both were included in the measurements of LV myocardial damaged volume by LGE, T2W and T1-mapping. Segments with MO were also analysed separately and delineated manually if simultaneously present on corresponding LGE, T1-mapping and T2W slices.

Wall motion abnormalities and LGE were assessed semi quantitatively by an experienced observer (E.D.) as described below. For regional function, segments were scored: 1 = normal; 2 = hypokinetic; 3 = akinetic or 4 = dyskinetic. The wall motion score index (WMSI = sum of segmental scores divided by the number of segments scored) was calculated as previously described [28]. The extent of LGE within each segment was estimated visually and categorized according to the percentage enhanced area of each segment (damaged area/segmental area) [29]: 0 = no LGE; 1 = 1 - 25% LGE; 2 = 26 - 50% LGE; 3 = 51 - 75% LGE and 4 = > 75%.

### Statistical Analyses

The normality was confirmed using the Kernell density plot. Mean (SD) values and median (interquartile range) were calculated for continuous variables. Student t-test was used for comparison of continuous variables collected at single time point (EF, WMSI, Troponin I). A random-effects linear regression model was used to describe the association between LGE, T2W (volumes of damaged measured as percentage) and T1 values (average relaxation time measured in msec.), controlling for clustering of segments within each subject. The overall fit of the model was investigated using χ^2 ^ratio test. Receiver operating characteristic (ROC) analysis was performed to identify cutoff values of T1 relaxation times and T2 SI ratio for detecting focal acute changes; this was achieved by using focal LGE scoring (LGE score ≥ 1) as the "true positive" surrogate marker for acute myocardial injury in this clinical setting, compared to segments with no LGE (LGE score = 0) in controls as the "true negative". Statistical significance of the differences between ROC curves was assessed using the method of DeLong et al.[30]. To analyze the presence of significant difference in T1 values in manually drawn ROIs in region of microvascular obstruction, ANOVA analyses were performed with Bonferroni post-hoc comparisons. A logistic regression model was used to predict the improvement of function at 6 months using as variables acute T1values and acute segmental damage fraction by LGE in matching patients with the same degree of damage based on LGE scoring. All statistical tests were two-tailed, and P-values of less than 0.05 were considered statistically significant.

## Results

Patient characteristics are presented in Table 1. Of the 43 patients enrolled, one patient could not complete the CMR protocol due to claustrophobia and one scan had to be excluded due to artefacts rendering the images non-analysable. Thus, a total of 41 patients (mean ± SD age, 56 ± 8 years, 78% with an ST elevation MI [STEMI]) were scanned within 24 hours post symptoms onset; out of these, 7 patients did not undergo the 6 m follow up scan (4 were excluded following CABG or staged PCI, and 3 refused to come back).

**Table 1:** Baseline characteristics of the study population

Patient characteristics	All N = 41	STEMI n = 32 (78%)	NSTEMI n = 9 (22%)	P-value
age	**56 ± 8**	56 ± 8	55 ± 8	**0.8**
sex	**3:38 (F:M)**	2:30 (F:M)	1:8 (F:M)	
Risk Factors [No (%)]				
Smoking	**14 (35)**	13 (40)	1 (11)	**0.13**
Hypertension	**15 (37)**	9 (28)	7 (78)	**0.17**
Diabetes	**4 (10)**	1 (3)	3 (34)	**0.03**
Family history	**15 (37)**	12(37)	3 (34)	**1.0**
Hyperlipidemia	**16 (40)**	10 (31)	5 (56)	**0.25**
Previous angina	**4**	2	2	**0.2**
Presenting characteristics				
Troponin I (12 hours post admission) (mg/mL)	**32 ± 20**	38 ± 15	9 ± 7	**< 0.01**
Median (IQR) Time from onset of symptoms to procedure (hrs)	**3 (2.2, 9)**	2.6 (2.0, 4.2)	24(13, 36)	**0.0001**
Culprit coronary artery [No (%)]				
LAD	**21 (52)**	17(53)	4 (44)	**1.0**
LCx	**4 (10)**	2 (6)	2 (22)	**0.3**
RCA	**17 (42)**	14 (40)	3 (33)	**0.15**
N of vessel diseased, n (%)				
0	**1**	0	1	**0.22**
1	**25 (61)**	24 (75)	1 (11)	**0.001**
2	**7 (17)**	5 (16)	3 (33)	**0.3**
3	**7 (17)**	3 (9)	4 (45)	**0.03**
Drug therapy n (%)				
Aspirin	**41 (100)**	32 (100)	9 (100)	**1**
Glycoprotein IIb/IIIa inhibitor	**33 (80)**	32 (100)	1 (11)	**< 0.001**
Clopidogrel	**41(100)**	32 (100)	9 (100)	**1**
**β**-Blockers	**41 (100)**	32 (100)	9 (100)	**1**
Heparin	**41 (100)**	32 (100)	9 (100)	**1**

Abbreviations: LAD- Left Anterior Descending; LCx - Left Circumflex; RCA- Right Coronary Artery;.

### CMR findings

The CMR findings are summarised in Table 2. All patients showed positive oedema and LGE. In acute setting, all patients had at least one LV dysfunctional segment; the global ejection fraction was at the lower end of normal (51% ± 11%). The relative mean volume of LGE in acute decreased significantly by 6M(P < 0.01) with a corresponding significant improvement in regional wall motion (P < 0.01).

**Table 2:** CMR findings

Variable	ALL (N = 41)	STEMI (N = 32)	NSTEMI (N = 9)	P-value (NSTEMI vs STEMI)
EF	48 ± 9	46 ± 8	58 ± 10	< 0.001
EDV	153 ± 36	157 ± 36	142 ± 39	0.2
ESV	81 ± 30	86 ± 30	65 ± 29	< 0.01
SV	73 ± 15	72 ± 15	79 ± 17	< 0.05
WMSI	1.6 ± 0.3	1.6 ± 0.3	1.4 ± 0.2	0.02
Oedema (T2W), LV% (Acute)	47 ± 14%	51 ± 12%	34 ± 10%	0.00015
LGE, LV % (acute)	38 ± 16%	41 ± 14%	25 ± 12%	0.0006
MO positive	7 (17%)	6 (18%)	1 (11%)	1
	**N = 34**	**N = 28**	**N = 6**	
EF (6M)	58 ± 11	56 ± 11	66 ± 3	0.1
WMSI (6M)	1.3 ± 0.2	1.3 ± 0.35	1.2 ± 0.2	0.2
LGE, LV% (6M)	28 ± 14%	30 ± 14%	21 ± 14%	0.1
Myocardial salvaged index (T2W)*	37 ± 27%	41 ± 23%	17 ± 37%	0.3

Abbreviations: EDV, end-diastolic volume; EF, Ejection fraction; ESV, end-systolic volume; LGE, late gadolinium enhancement; LV, left ventricle;MO, microvascular obstruction; SV, stroke volume; T2W, T2 weighted; WMSI, wall motion score index.* myocardial salvage index was calculated using the final infarct size at 6 months (6M)

### T1 values in areas of acute myocardial injury by LGE and T2W

**Figure 1:**
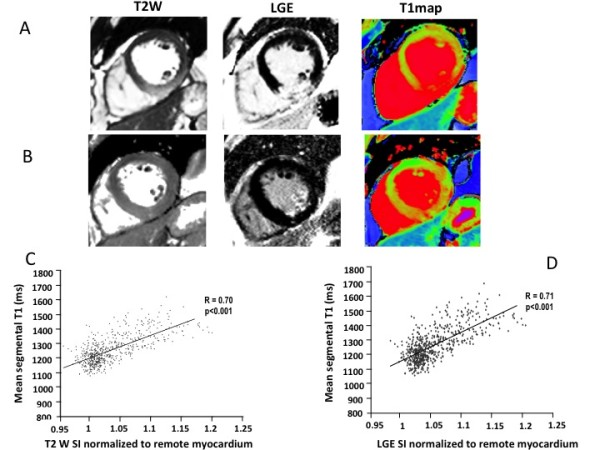
**Panels A and B. Representative CMR images**. Oedema T2W images (left column), acute LGE images (center), and ShMOLLI T1-mapping (right column) are displayed. Two sets of images (Panel A and Panel B) corresponding to two separate patients are shown. Top panels (A): a case of transmural inferior STEMI:both oedema (T2W) and LGE depict an area of increased signal intensity; in the same region T1-mapping depicts significantly increased T1 values (shown in red) compared to the remote unaffected myocardium (normal T1 values shown in green). Lower panels (B), a case of subendocardial NSTEMI : Although the T2 W images show only a mild increase in brightness, there is an area of increased T1 values exceeding the area of LGE enhancement. Of note the peak troponin I was significantly different in the two patients (peak troponin I 50 mg/mL in the STEMI patient vs7 mg/mL in the NSTEMI patient). Panels C and D: Correlation between T1 values and T2W normalized SI (Panel C) and between T1 values and LGE normalized SI (Panel D). The SI of T2W (on the × axis, panel C) and LGE (on × axis Panel D), both normalized to the remote unaffected myocardium, are shown to correlate strongly with T1 values.

Representative examples of T2W, LGE and T1-mapping from a STEMI and a NSTEMI patient are shown in Figure 1, Panel A and B respectively. Increased T1 values are shown in areas of myocardium co-localized with areas of LGE and increased signal on T2W-CMR; in the NSTEMI patient T1values are increased in an area larger then the one enhanced by LGE.

In order to investigate the accuracy of T1 measurements in comparison to T2W, we first assessed the variability of the T1-values in remote unaffected myocardial segments with no ischemic injury (as confirmed by LGE, T2W or wall motion) and compared it to the signal intensities in T2W imaging of corresponding segments. The average T1 value (± SD) in unaffected myocardium was the same for STEMI and NSTEMI (1189 ± 60 msec and 1176 ± 38 msec respectively, P = 0.16), with values similar to volunteers at 3T [17] (1166 ± 60 msec)with a coefficient of variation (CV) significantly lower compared to T2W (6% versus 14% respectively, P < 0.0001).

In order to assess the relationship between areas of injury depicted byT2W or LGE and areas of increased T1 values on T1-mapping, we then investigated the correlation between absolute T1 values and the signal intensity of LGE and/orT2W on a segmental basis. As shown in Figure 1 Panels C and D, increased signal intensity on LGE images correlated with increased absolute T1 values (r = 0.71, P < 0.001) and with T2W signal intensities (r = 0.65, P < 0.001) in matching segments, indicating co-localization of injured myocardium. Similarly, a strong correlation was shown between T1values andT2W signal intensities, with T1 values ranging between 1078 msec and 1624 msec (1257 ± 97 msec - mean ± SD) in the acutely ischemic segments. The T1 measurements in acutely ischemic segments were significantly different compared to T1 values in normal unaffected segments (1257 ± 97 msec vs 1196 ± 56 msec, P < 0.01).

### Diagnostic performance of T1-mapping in detecting acute injury in acute myocardial infarction

**Figure 2:**
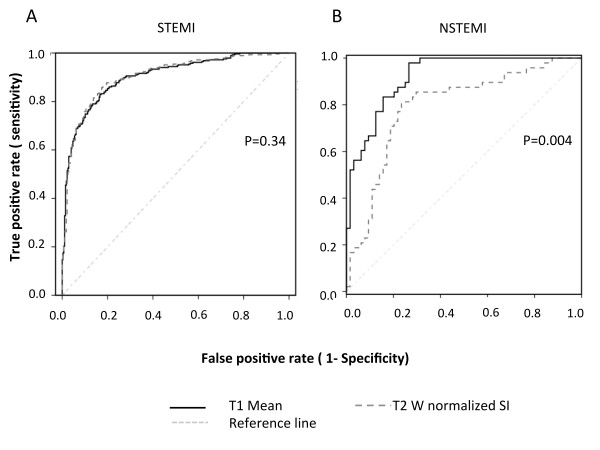
**Receiver Operating Curves showing the diagnostic performance of T1-mapping compared toT2W in detecting acute injury in ACS as derived from late gadolinium enhancement (LGE) scoring**. The area under the curve forT2W (dashed line) was not significantly different from T1-mapping (solid line) in patients with STEMI (Panel A); In NSTEMI patients, the area under the curve forT2W (dashed line) was significantly smaller than that for T1-mapping (p = 0.004).

Figure 2 illustrates the diagnostic performance of T1-mapping compared to the T2W technique in detecting acute myocardial injury against acute LGE manual scoring as the gold standard based on ROC analysis. In MI patients, both techniques performed equally (T1-mapping area under the curve 0.90 ± 0.01 vs T2W area under the curve 0.89 ± 0.01, P = 0.34) (Figure 2, Panel A). In order to investigate whether the diagnostic performance of T1-mapping could be better in patients with smaller infarcts, we performed ROC analyses in the subgroup of NSTEMI patients. In these patients T1-mapping showed improved detection of myocardial injury compared to T2W (T1-mapping area under the curve 0.91 ± 0.02 vs T2W area under the curve 0.81 ± 0.04, P = 0.004).

### Relation between absolute T1 values and the extent of myocardial injury

**Figure 3:**
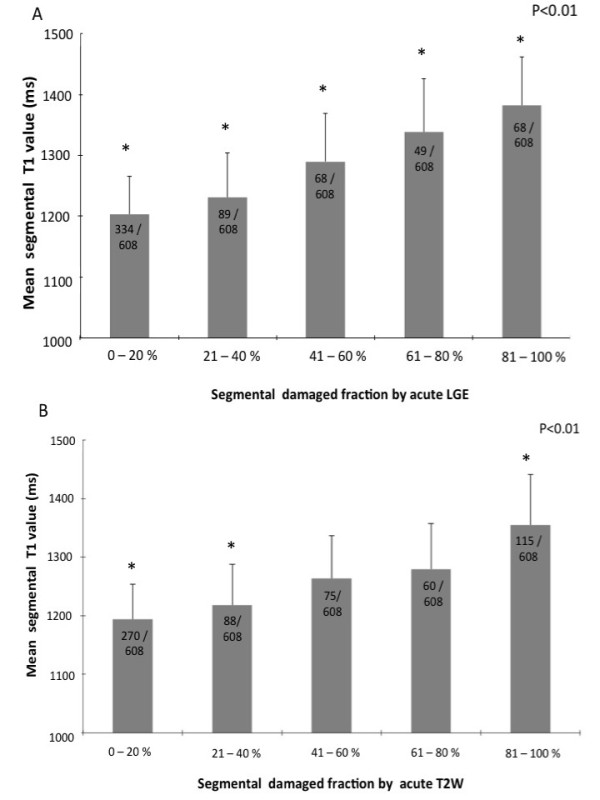
**Assessment of severity of injury by T1-mapping: relationship withT2W and LGE**. T1 values increase for increasing segmental extent of injury, assessed by either LGE (panel A) or T2W (panel B). The overall relationships are significant: however for T2W, there is an overlap in T1 values for segments with segmental damaged fraction between 41 and 80%. Lesions with MO have been excluded for this analysis.

In order to explore the potential of quantitative T1-mapping to assess the extent of acute ischemic injury on a segmental basis against the current gold standards of LGE and oedema imaging, we investigated the relationship between average segmental T1 values and the volumetric lesion fraction (by LGE andT2W) (Figure 3 Panel A and B). For this analysis, segments with MO were excluded (see below paragraph onT1s in MO). A significant relationship between the segmental extent of myocardial injury as defined by LGE and T1 values was found (P < 0.001). Increasing T1 values clearly distinguished 20% increments in segmental LGE fractions A significant relationship was also found between segmental fractions of oedema assessed byT2W and T1 values (P < 0.001); however, there was an overlap in T1 values in segments with intermediate extent of oedema (41% to 80% segmental fraction).

**Figure 4:**
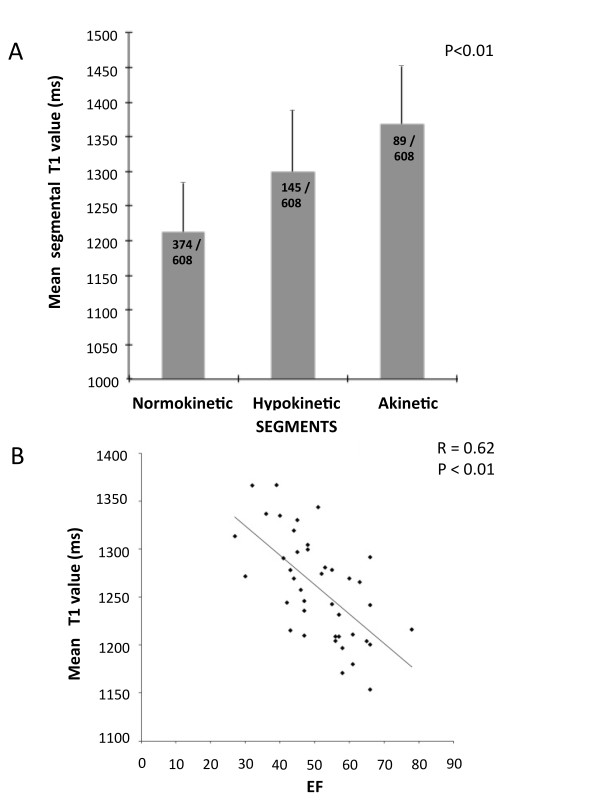
**Assessment of severity of injury by T1-mapping: relationship with regional and global function**. Panel A shows the relationship between T1 values and the extent of regional wall motion abnormalities (P < 0.01). A highly significant negative correlation between mean LV T1 values and LV ejection fraction was also found (Panel B).

To investigate whether quantitative characterization of myocardial tissue in MI reflects not only myocardial damage by LGE but also functional contractile impairment, we assessed the correlation between T1 values and both regional and global systolic function (Figure 4, Panel A and B).

A significant relationship (P < 0.01) was found between T1 values and regional wall motion impairment: while T1 values were within normal range in normally functioning segments (1196 ± 85 msec) they increased significantly in hypokinetic segments (1299 ± 90 msec), reaching maximal values (1368 ± 83 msec) in akinetic segments. Furthermore, we found a highly significant correlation between global myocardial T1 values and LV ejection fraction (P < 0.01)

### Salvaged myocardium assessment byT1-mapping

In order to match, on a like-for-like basis, the relative LV mass of acutely damaged myocardium by T1-mapping with the one assessed by T2W and to derive comparable values of salvaged myocardium, we used a threshold of 10% (close to 2 standard deviations) above the previously established T1 values in normal controls [17]. By empirically choosing 10%above the mean normal T1, the relative LV mass of acutely damaged myocardium was 43 ± 19% by T1-mapping versus 47 ± 14% by T2W, P = 0.2. The derived salvaged index using the final infarct size by LGE at 6 months was 37 ± 24% by T1-mapping versus 37 ± 27% by T2W (P = 0.88).

### Characterization of microvascular obstruction by T1-mapping

Overall, 7patients showed evidence of MO on LGE imaging (6 with STEMI and 1 NSTEMI).

**Figure 5:**
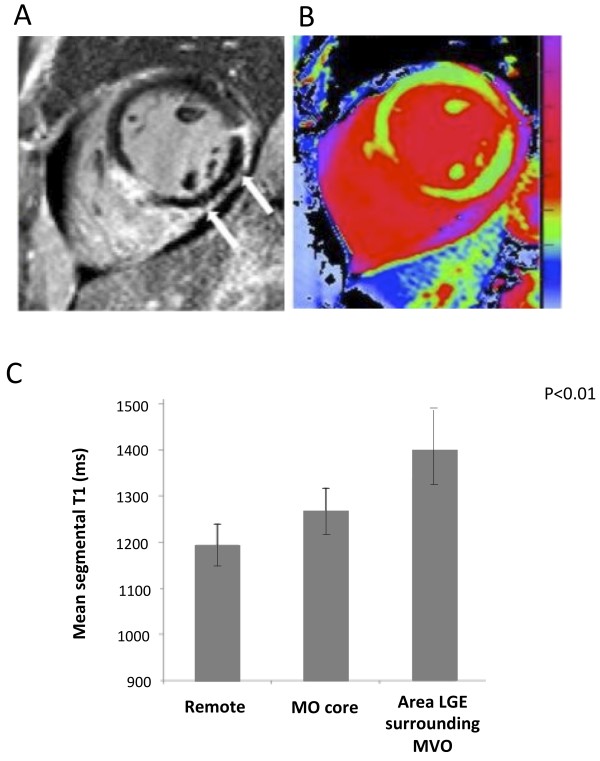
**Quantitative assessment of microvascular obstruction by T1-mapping**. Panel A shows a case of microvascular obstruction as depicted by LGE. The corresponding T1-map is shown in Panel B. The dark core of MO in the LGE image (arrows) is depicted by T1 values (Panel B) in the color range similar to remote myocardium (green). The surrounding injured myocardium (bright on LGE) shows high T1 values (red). Panel C shows quantitative analyses of the 7 cases with MO. T1 values in the MO regions are significantly lower than in the surrounding LGE-positive myocardium.

A typical case is shown in Figure 5. On average, T1 values in the core of MO were significantly higher than in the remote myocardium (1267 ± 52 msec vs1194 ± 47 msec, P = 0.002), but significantly lower than in the surrounding injured myocardium within the LGE positive myocardium (1403 ± 80 msec, P < 0.01 versus remote myocardium and MO) (Figure 5, Panel C).

### Likelihood of functional recovery at 6 months by acute T1-mapping

**Figure 6:**
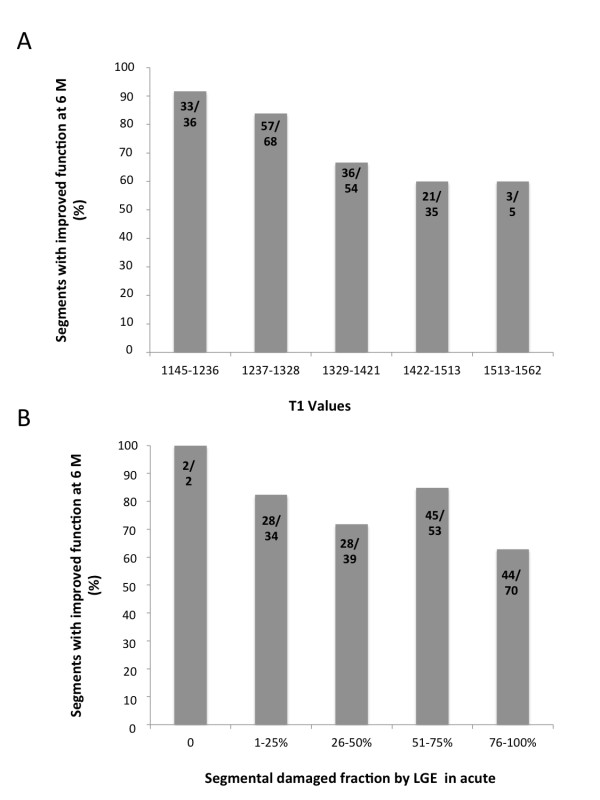
**Likelihood of functional improvement at 6 m by T1-mapping and acute LGE in dysfunctional segments**. Data are shown for 198 segments, which were dysfunctional acutely. Panel A shows the significant relationship between the likelihood of recovery of function of acutely dysfunctional segments and the T1 values in acute (P < 0.0004). Panel B shows the relationship between the likelihood of recovery of function of acutely dysfunctional segments and the segmental damaged fraction by LGE in acute (P < 0.004). While the relationship between functional recovery and T1 was essentially linear, by using acute LGE (Panel B) the likelihood of improvement for segments with intermediate LGE did not correlate with the amount of damage. No MO segments were included in this analysis.

T1 values correlate with the extent of segmental LGE in the acute setting. In order to assess whether acute T1 values predict functional recovery at 6 months, we assessed the ability of acute T1 values and of the acute LGE segmental fraction to predict the likelihood of long-term segmental wall motion improvement in 198 acutely dysfunctioning segments (excluding MO). As shown in Figure 6, Panel A, there was a significant relationship between acute T1 values and the improvement of function at 6 months (P < 0.0004): virtually all dysfunctional segments with only slightly increased T1s in acute setting recovered function at 6M, while for values higher than 1406 msec, the percentage of segments which improved wall motion at 6M decreased to 60%. The relationship between acute LGE and improvement of function was also significant overall (P < 0.004). However, while the relationship between functional recovery and T1 was essentially linear, by using acute LGE (Figure 6, Panel B), the likelihood of improvement for those segments with intermediate LGE (between 25% and 75%), did not correlate with the amount of damage.

## Discussion

The main findings of our study are: 1) quantitative T1-mapping is a more robust technique compared to T2W, as T1 measurements show a significantly lower variability than T2W, their acquisition is faster with a better spatial resolution (reconstructed pixel size of 0.9 × 0.9 mm/experimental pixel size 1.8 × 1.8 mm versus 2.1 × 1.6 mm) 2) non contrast T1-mapping accurately detect acutely injured myocardium in MI patients when compared to T2W and LGE as markers of acute injury; 3) the diagnostic performance of T1-mapping is at least as good as that of T2W-CMR for detecting acute myocardial injury, 4) non-contrast T1-mapping allows assessment of the extent of acute myocardial damage on a segmental level 5) myocardial salvage index can be calculated based on T1-mapping with similar results compared to T2W 6) T1 values are strongly related to the likelihood of functional improvement at 6 months.

We believe that the results of this study are of direct clinical relevance. For the first time we report on the potential use of quantitative pre-contrast T1-mapping techniques to further improve our ability to characterize the presence and extent of acute myocardial injury and to determine reversible myocardial injury and salvaged myocardium by CMR. The current CMR imaging techniques used to assess acute ischemic injury, T2W and LGE imaging, have important limitations in relation to: a) their clinical applicability in acutely ill patients not always capable to hold their breath; b) the accurate assessment of the injury due to inherent issues such as variability of the measurements because of image gradients, partial volume effects and threshold dependent postprocessing. T1-mapping allows for faster acquisition compared to standard T2W and LGE techniques, and it is a more robust technique with less variability in T1 measurements at improved spatial resolution. Furthemore, both LGE and T2W are semi-quantitative methods which rely on the use of an arbitrary threshold, usually set at 2 SD above remote 'normal' myocardium, to delineate areas of myocardial oedema and/or scarring [14]. However, the use of a reference ROI in the remote myocardium may lead to false negative findings when systemic processes globally affect the heart. Secondly, it is unclear whether this arbitrary threshold of 2 SD (rather than 3 SD or 5 SD) accurately reflects myocardial tissue changes occurring early after myocardial infarction [14]. Thirdly, post processing is time-consuming and not always suitable to applications in acute setting. We recently showed that, due to the dynamic pathophysiological tissue changes occurring very early after an acute ischemic event, there is a significant variability in acute CMR imaging findings, and consequently in derived clinical prognostic indices such as salvageable myocardium [6]. These issues often make the interpretation of CMR findings in acute MI difficult. A novel CMR technique, which would allow for a more detailed and quantitative characterization of acutely injured myocardium, would thus be highly desirable. Our findings suggest that quantitative T1-mapping techniques are a suitable tool for this. A recently validated improved technique (ShMOLLI)[17] allows for fast acquisition of high resolution pre-contrast T1-mapseven at patients with high heart rates and therefore it is well suited for imaging ACS patients [17].

This is the first study in patients with ACS that has used T1-mapping at 3 Tesla to assess the presence and the extent of acute ischemic injury compared to accepted gold standard measurements of salvageable myocardium such as T2W and LGE-CMR. To the best of our knowledge, no other T1-mapping studies have been conducted at 3T; however previous work conducted at 1.5T CMR by Messroghli et al.[21] demonstrated that pre-contrast T1-mapping can detect acute myocardial infarction with high sensitivity and specificity (96% and 91%, respectively), against LGE as the gold standard. Goldfarb et al.[20] also showed increased T1 values in LGE positive myocardial infarcts up to 1 month after the acute event. However, in both these studies, oedema imaging by T2W-CMR was not performed [20,21]. As shown in previous animal studies, the mechanism underlying the increase in T1 values in acutely ischemic myocardium is likely related to the increase in tissue water content, which has been shown to exceed the area of infarction by histological assessment [19,31]. We confirm these experimental findings in the clinical setting, as we demonstrated higher T1 values in areas of oedematous myocardium within 24 hours after an acute ischemic event. Our results also indicate that quantitative T1-mapping measurements correlate well withT2W measurements, but show significantly lower variability than T2W-CMR. Therefore, T1-mapping may be superior to T2W-CMR for assessment of acute myocardial injury in MI. Indeed, this is further supported by the better diagnostic performance of T1-mapping compared to T2W-CMR in detecting small areas of acute myocardial injury in patients with NSTEMI.

Our results also indicate that T1-mapping allows for the assessment of the extent of *segmental *injury in the acute setting. This is further supported by the relationship between T1 values and contractile function. The impairment of contractile function in regions with high T1 values is due to severe myocardial damage. The assessment of irreversible injury in the early days following an acute ischemic event is challenging due to several issues. Recently published data,[6-9] showed a rapid reduction in LGE volumes occurring in the days following the acute event and have therefore challenged the view that acute LGE equals to stable irreversible injury but may rather reflect a dynamic process resulting from. Therefore, acute LGE (within 7 days of acute MI) might not be a reliable predictor of functional recovery in this setting. Furthermore, Matsumoto et al.[10] recently validated early gadolinium enhancement imaging to assess area at risk, indicating that the imaging time to assess irreversible injury acutely is critical and poorly defined. Others have suggested that, to obtain an accurate assessment of the irreversible injury, late gadolinium should be acquired 20 minutes post injection [32] with consequent prolongation of the acquisition protocol and limited applicability in unstable acutely infarcted patients. Based on our findings, high T1 values in acutely ischemic myocardium reflect both, areas positive for T2W and for LGE. Previous reports in experimental models have shown that, following prolonged ischemia, T1 values may increase above and beyond the corresponding increase in water content; this may be related to a more severe degree of cellular injury with consequent release of intracellular ions into the extracellular space [19]. In keeping with these experimental findings, our results indicate that the likelihood of improvement in function at 6 months *decreases *with incremental increases of T1 values. Based on these results, T1-mapping may be considered a better predictor of recovery of function compared to acute LGE although the predictive value of T1-mapping does not reach the predictive value of chronic LGE [29]. Clearly, acute T1-mapping reflects the sum of reversible and irreversible injury while chronic LGE represents irreversible injury only, therefore a better relationship with 6 months functional improvement is expected for the latter [29,33]. However, information on chronic LGE findings is not available when assessing patients acutely. Further studies will be needed to assess the potential of T1-mapping to depict the changes occurring in the myocardium in the early days post MI and their predictive value.

Finally, we demonstrated that T1 values are significantly lower in areas of MO compared to T1 values in infarcted areas without MO. This is likely to a combination of blood degradation products (hemorrhage) and reduced water content, which both result in lower T1 values compared to infarcts without MO. The prognostic value of MO/hemorrhage as a predictor of adverse LV remodeling [34,35] is well recognized. Further work is needed to better characterize areas of MO with non-contrast T1-mapping and to determine whether additional prognostic information can be derived by ShMOLLI T1-mapping in this setting.

### Limitations

Even though T1-mapping is an inherently quantitative method providing pixel-wise absolute T1 values, the lack of invasive tissue sampling only permits comparisons between arbitrary chosen thresholds. We chose 1271 msec as the closest value to replicate the common 2 SD threshold used in T2W oedema imaging. The use of an arbitrary choice is unavoidable in signal-intensity based imaging methods and it makes the method robust to different field strength and acquisition sequences [17]. However, a 10% threshold might still change, and further investigation (possibly with histologic comparison) will be needed to fully address this issue.

Also, at 3T we encountered frequent SSFP off-resonance artifacts despite shim correction, and 10% of ShMOLLI segments had to be excluded from analysis. This limitation is strictly related to high-field imaging: in fact the incidence of these artifacts is expected to be lower at 1.5Tallowing for application in clinical routine practice. Secondly, T2 mapping is a promising new CMR technique for assessing acute myocardial injury [36], which was not included in this study. We did, however, compare our T1-mapping approach with a novel, state-of-the-art T2prep-SSFP sequence shown to be superior to traditional T2W STIR imaging and widely considered a gold standard for oedema imaging in the acute setting. Thirdly, the 6 months follow up was not performed in 17% of our acute population. Although a complete follow up would have been desirable, the final sample size used to assess the relationship between T1 values in acute and long term functional improvement was adequate based on 80% power calculation with an accuracy of 0.05. Finally, we do not provide histopathological confirmation of our findings and the precise mechanisms leading to increase T1 values in acute myocardial injury warrant further investigation.

## Conclusion

Increased pre-contrast T1 values depict areas of acutely injured myocardium with a diagnostic accuracy at least as good if not superior to T2W. In patients with acute MI, incremental increases in T1 values delineate the severity of the extent myocardial injury and predict functional recovery at 6 months. Although further investigations will be needed, our results suggest that pre-contrast T1-mapping may represent a valuable alternative to standard oedema and scar imaging methods with particular usefulness in the assessment of reversible myocardial injury and salvaged myocardium with no need for post-processing.

## Abbreviations

CABG: coronary artery bypass grafts; CMR: Cardiovascular magnetic Resonance; CV: coefficient of variation; EF: ejection fraction; LGE: late gadolinium enhancement; LV: left ventricle; MI: Myocardial infarction; MO: microvascular obstruction; NSTEMI: non ST elevation MI; PCI: percutaneous coronary intervention; ShMOLLI: Shortened Modified Look-Looker Inversion recovery; STEMI: ST elevation myocardial infarction; T2W: T2 weighted

## Competing interests

The authors declare that they have no competing interests.

## Authors' contributions

EDA: contributed substantially to conception and design of study; acquired and analyzed the data; drafted the manuscript; SKP: contributed substantially to data analyses; VMF: contributed to data acquisition and analyses, critical revision of the manuscript; QLS: contributed to statistical analyses; MDR: have made substantial contribution to the deign and critically revised the manuscript; JMF and FC: have contributed to data acquisition; RKK, APB, RPC, and TK: have critically revised the manuscript; SN: conceived the study, participated in its design and coordination and helped to draft the manuscript. All authors read and approved the final manuscript.

## Supplementary Material

Additional File 1 **CMR protocol**. This additional figure shows the CMR protocol used. Four different CMR techniques were included: T2W imaging for assessment of edema, T1 mapping, functional cine imaging and late gadolinium enhancement for assessment of necrosis. Following the acquisition of pilot and long axis images, matching short axis slices covering the full length of the ventricle were acquired using each of the different techniques. T2W and T1 mapping acquisitions were performed prior to the administration of contrast. Following gadolinium, cine imaging was performed. Ten-fifteen minutes post contrast and after the inversion time was meticulously adjusted for optimal nulling of remote normal myocardium, late gadolinium enhancement imaging was completed. Click here for file

## References

[B1] Lima JAC Judd RM Bazille A Schulman SP Atalar E Zerhouni EA Regional Heterogeneity of Human Myocardial Infarcts Demonstrated by Contrast-Enhanced MRI: Potential Mechanisms Circulation 1995 92 1117 1125 7648655 10.1161/01.cir.92.5.1117

[B2] Choi KM Kim RJ Gubernikoff G Vargas JD Parker M Judd RM Transmural extent of acute myocardial infarction predicts long-term improvement in contractile function Circulation 2001 104 1101 1107 10.1161/hc3501.096798 11535563

[B3] Aletras AH Tilak GS Natanzon A Hsu L-Y Gonzalez FM Hoyt RF Arai AE Retrospective Determination of the Area at Risk for Reperfused Acute Myocardial Infarction With T2-Weighted Cardiac Magnetic Resonance Imaging: Histopathological and Displacement Encoding With Stimulated Echoes (DENSE) Functional Validations Circulation 2006 113 1865 1870 10.1161/CIRCULATIONAHA.105.576025 16606793

[B4] Abdel-Aty H Cocker M Meek C Tyberg JV Friedrich MG Edema as a very early marker for acute myocardial ischemia: a cardiovascular magnetic resonance study J Am Coll Cardiol 2009 53 1194 1201 10.1016/j.jacc.2008.10.065 19341860

[B5] Friedrich MG Abdel-Aty H Taylor A Schulz-Menger J Messroghli D Dietz R The salvaged area at risk in reperfused acute myocardial infarction as visualized by cardiovascular magnetic resonance J Am Coll Cardiol 2008 51 1581 1587 10.1016/j.jacc.2008.01.019 18420102

[B6] Dall'Armellina E Karia N Lindsay AC Karamitsos TD Ferreira V Robson MD Kellman P Francis JM Forfar C Prendergast BD Banning AP Channon KM Kharbanda RK Neubauer S Choudhury RP Dynamic Changes of Edema and Late Gadolinium Enhancement after Acute Myocardial Infarction and Their Relationship to Functional Recovery and Salvage Index Circulation: Cardiovascular Imaging 2011 4 228 236 10.1161/CIRCIMAGING.111.963421 PMC3098134 21447711

[B7] Engblom H Rapid initial reduction of hyperenhanced myocardium after reperfused first myocardial infarction suggests recovery of the peri-infarction zone: one year follow up by MRI Circulation cardiovascular imaging 2009 2 47 55 10.1161/CIRCIMAGING.108.802199 19808564

[B8] Ibrahim T Makowski MR Jankauskas A Maintz D Karch M Schachoff S Manning WJ Schomig A Schwaiger M Botnar RM Serial Contrast-Enhanced Cardiac Magnetic Resonance Imaging Demonstrates Regression of Hyperenhancement Within the Coronary Artery Wall in Patients After Acute Myocardial Infarction J Am Coll Cardiol Img 2009 2 580 588 10.1016/j.jcmg.2008.12.029 19442944

[B9] Kramer CM Rogers WJ Jr Mankad S Theobald TM Pakstis DL Hu YL Contractile reserve and contrast uptake pattern by magnetic resonance imaging and functional recovery after reperfused myocardial infarction Journal of the American College of Cardiology 2000 36 1835 1840 10.1016/S0735-1097(00)00945-1 11092653

[B10] Matsumoto H Matsuda T Miyamoto K Shimada T Mikuri M Hiraoka Y Peri-Infarct Zone on Early Contrast-Enhanced CMR Imaging in Patients With Acute Myocardial Infarction JACC: Cardiovascular Imaging 2011 4 610 618 10.1016/j.jcmg.2011.03.015 21679895

[B11] Friedrich MG Kim HW Kim RJ T2-Weighted Imaging to Assess Post-Infarct Myocardium at Risk JACC: Cardiovascular Imaging 2011 4 1014 1021 10.1016/j.jcmg.2011.07.005 21920341 PMC3206638

[B12] Kellman P Aletras AH Mancini C McVeigh ER Arai AE T2-prepared SSFP improves diagnostic confidence in edema imaging in acute myocardial infarction compared to turbo spin echo Magn Reson Med 2007 57 891 897 10.1002/mrm.21215 17457880 PMC2396276

[B13] Payne AR Casey M McClure J McGeoch R Murphy A Woodward R Saul A Bi X Zuehlsdorff S Oldroyd KG Tzemos N Berry C Bright Blood T2 Weighted MRI Has Higher Diagnostic Accuracy Than Dark Blood STIR MRI for Detection of Acute Myocardial Infarction and for Assessment of the Ischemic Area-at-Risk and Myocardial Salvage Circulation: Cardiovascular Imaging 2011 4 210 219 10.1161/CIRCIMAGING.110.960450 21427362

[B14] Wince WB Kim RJ Molecular imaging: T2-weighted CMR of the area at risk-a risky business? Nature reviews Cardiology 2010 7 547 549 10.1038/nrcardio.2010.124 20865026

[B15] Kwong RY Farzaneh-Far A Measuring Myocardial Scar by CMR JACC Cardiovascular imaging 2011 4 157 160 10.1016/j.jcmg.2010.12.004 21329900

[B16] Messroghli DR Radjenovic A Kozerke S Higgins DM Sivananthan MU Ridgway JP Modified Look-Locker inversion recovery (MOLLI) for high-resolution T1 mapping of the heart Magnetic resonance in medicine: official journal of the Society of Magnetic Resonance in Medicine/Society of Magnetic Resonance in Medicine 2004 52 141 146 10.1002/mrm.20110 15236377

[B17] Piechnik S Ferreira V Dall'Armellina E Cochlin L Greiser A Neubauer S Robson M Shortened Modified Look-Locker Inversion recovery (ShMOLLI) for clinical myocardial T1-mapping at 1.5 and 3 T within a 9 heartbeat breathhold Journal of Cardiovascular Magnetic Resonance 2010 12 69 10.1186/1532-429X-12-69 21092095 PMC3001433

[B18] Higgins CB Herfkens R Lipton MJ Sievers R Sheldon P Kaufman L Crooks LE Nuclear magnetic resonance imaging of acute myocardial infarction in dogs: alterations in magnetic relaxation times Am J Cardiol 1983 52 184 188 10.1016/0002-9149(83)90093-0 6858909

[B19] Williams ES Kaplan JI Thatcher F Zimmerman G Knoebel SB Prolongation of proton spin lattice relaxation times in regionally ischemic tissue from dog hearts Journal of nuclear medicine: official publication, Society of Nuclear Medicine 1980 21 449 453 7373415

[B20] Goldfarb JW Arnold S Han J Recent myocardial infarction: assessment with unenhanced T1-weighted MR imaging Radiology 2007 245 245 250 10.1148/radiol.2451061590 17885192

[B21] Messroghli DR Walters K Plein S Sparrow P Friedrich MG Ridgway JP Sivananthan MU Myocardial T1 mapping: application to patients with acute and chronic myocardial infarction Magnetic resonance in medicine: official journal of the Society of Magnetic Resonance in Medicine/Society of Magnetic Resonance in Medicine 2007 58 34 40 10.1002/mrm.21272 17659622

[B22] Kushner FG Hand M Smith SC Jr King SB III Anderson JL Antman EM Bailey SR Bates ER Blankenship JC Casey DE Jr 2009 Focused Updates: ACC/AHA Guidelines for the Management of Patients With ST-Elevation Myocardial Infarction (Updating the 2004 Guideline and 2007 Focused Update) and ACC/AHA/SCAI Guidelines on Percutaneous Coronary Intervention (Updating the 2005 Guideline and 2007 Focused Update): A Report of the American College of Cardiology Foundation/American Heart Association Task Force on Practice Guidelines Circulation 2009 120 2271 2306 10.1161/CIRCULATIONAHA.109.192663 19923169

[B23] Anderson JL Adams CD Antman EM Bridges CR Califf RM Casey DE Jr Chavey WE Fesmire FM Hochman JS Levin TN ACC/AHA 2007 guidelines for the management of patients with unstable angina/non-ST-Elevation myocardial infarction: a report of the American College of Cardiology/American Heart Association Task Force on Practice Guidelines (Writing Committee to Revise the 2002 Guidelines for the Management of Patients With Unstable Angina/Non-ST-Elevation Myocardial Infarction) developed in collaboration with the American College of Emergency Physicians, the Society for Cardiovascular Angiography and Interventions, and the Society of Thoracic Surgeons endorsed by the American Association of Cardiovascular and Pulmonary Rehabilitation and the Society for Academic Emergency Medicine J Am Coll Cardiol 2007 50 e1 e157 17692738 10.1016/j.jacc.2007.02.013

[B24] Kellman P Arai AE McVeigh ER Aletras AH Phase-sensitive inversion recovery for detecting myocardial infarction using gadolinium-delayed hyperenhancement Magnetic resonance in medicine: official journal of the Society of Magnetic Resonance in Medicine/Society of Magnetic Resonance in Medicine 2002 47 372 383 10.1002/mrm.10051 PMC2041905 11810682

[B25] Karamitsos TD Hudsmith LE Selvanayagam JB Neubauer S Francis JM Operator induced variability in left ventricular measurements with cardiovascular magnetic resonance is improved after training J Cardiovasc Magn Reson 2007 9 777 783 10.1080/10976640701545073 17891615

[B26] Berry C Kellman P Mancini C Chen MY Bandettini WP Lowrey T Hsu LY Aletras AH Arai AE Magnetic Resonance Imaging Delineates the Ischemic Area-at-Risk and Myocardial Salvage in Patients with Acute Myocardial Infarction Circ Cardiovasc Imaging 2010 3 527 535 10.1161/CIRCIMAGING.109.900761 20631034 PMC2966468

[B27] Kim RJ Fieno DS Parrish TB Harris K Chen E-L Simonetti O Bundy J Finn JP Klocke FJ Judd RM Relationship of MRI Delayed Contrast Enhancement to Irreversible Injury, Infarct Age, and Contractile Function Circulation 1999 100 1992 2002 10556226 10.1161/01.cir.100.19.1992

[B28] Dall'Armellina E Morgan TM Mandapaka S Ntim W Carr JJ Hamilton CA Hoyle J Clark H Clark P Link KM Case D Hundley WG Prediction of Cardiac Events in Patients With Reduced Left Ventricular Ejection Fraction With Dobutamine Cardiovascular Magnetic Resonance Assessment of Wall Motion Score Index Journal of the American College of Cardiology 2008 52 279 286 10.1016/j.jacc.2008.04.025 18634983 PMC3666037

[B29] Kim RJ Wu E Rafael A Chen EL Parker MA Simonetti O Klocke FJ Bonow RO Judd RM The use of contrast-enhanced magnetic resonance imaging to identify reversible myocardial dysfunction N Engl J Med 2000 343 1445 1453 10.1056/NEJM200011163432003 11078769

[B30] DeLong ER DeLong DM Clarke-Pearson DL Comparing the areas under two or more correlated receiver operating characteristic curves: a nonparametric approach Biometrics 1988 44 837 845 10.2307/2531595 3203132

[B31] Brown JJ Peck WW Gerber KH Higgins CB Strich G Slutsky RA Nuclear magnetic resonance analysis of acute and chronic myocardial infarction in dogs: alterations in spin-lattice relaxation times American Heart Journal 1984 108 1292 1297 10.1016/0002-8703(84)90756-7 6496288

[B32] Andrew EA Gadolinium Can Depict Area at Risk and Myocardial Infarction: A Double-Edged Sword? JACC: Cardiovascular Imaging 2011 4 619 621 10.1016/j.jcmg.2011.04.006 21679896 PMC3753673

[B33] Selvanayagam JB Kardos A Francis JM Wiesmann F Petersen SE Taggart DP Neubauer S Value of delayed-enhancement cardiovascular magnetic resonance imaging in predicting myocardial viability after surgical revascularization Circulation 2004 110 1535 1541 10.1161/01.CIR.0000142045.22628.74 15353496

[B34] Wu KC Zerhouni EA Judd RM Lugo-Olivieri CH Barouch LA Schulman SP Blumenthal RS Lima JAC Prognostic Significance of Microvascular Obstruction by Magnetic Resonance Imaging in Patients With Acute Myocardial Infarction Circulation 1998 97 765 772 9498540 10.1161/01.cir.97.8.765

[B35] Mather AN Fairbairn TA Ball SG Greenwood JP Plein S Reperfusion haemorrhage as determined by cardiovascular MRI is a predictor of adverse left ventricular remodelling and markers of late arrhythmic risk Heart 2011 97 453 459 10.1136/hrt.2010.202028 21051455

[B36] Giri S Chung YC Merchant A Mihai G Rajagopalan S Raman SV Simonetti OP T2 quantification for improved detection of myocardial edema J Cardiovasc Magn Reson 2009 11 56 10.1186/1532-429X-11-56 20042111 PMC2809052

