# Supplementary material for: Cardiovascular magnetic resonance by non contrast T1-mapping allows assessment of severity of injury in acute myocardial infarction
Source: J Cardiovasc Magn Reson. 2012 Feb 6;14(1):15. doi: 10.1186/1532-429X-14-15 (PMC3312869; doi:10.1186/1532-429X-14-15)
Supplement: Additional File 1 — CMR protocol. This additional figure shows the CMR protocol used. Four different CMR techniques were included: T2W imaging for assessment of edema, T1 mapping, functional cine imaging and late gadolinium enhancement for assessment of necrosis. Following the acquisition of pilot and long axis images, matching short axis slices covering the full length of the ventricle were acquired using each of the different techniques. T2W and T1 mapping acquisitions were performed prior to the administration of contrast. Following gadolinium, cine imaging was performed. Ten-fifteen minutes post contrast and after the inversion time was meticulously adjusted for optimal nulling of remote normal myocardium, late gadolinium enhancement imaging was completed. [file 1532-429X-14-15-S1.DOCX]

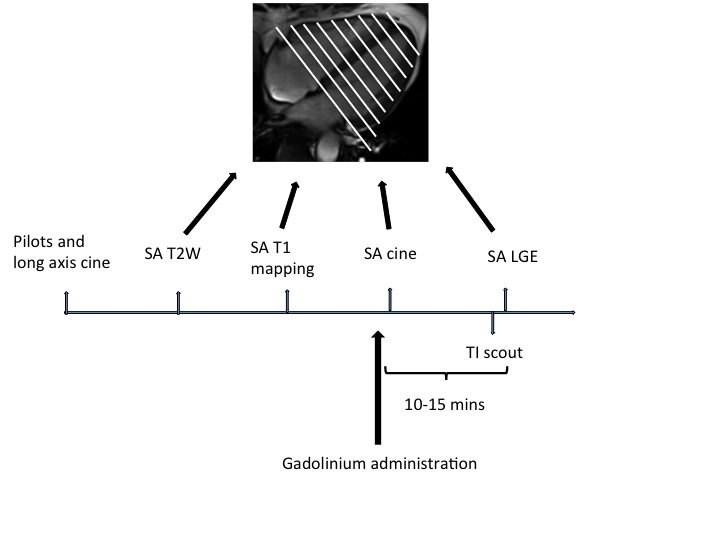


**Additional File 1**

**CMR protocol.** Four different CMR techniques were included: T2W imaging for assessment of edema, T1 mapping, functional cine imaging and late gadolinium enhancement for assessment of necrosis. Following the acquisition of pilot and long axis images, matching short axis slices covering the full length of the ventricle were acquired using each of the different techniques. T2W and T1 mapping acquisitions were performed prior to the administration of contrast. Following gadolinium, cine imaging was performed. Ten-fifteen minutes post contrast and after the inversion time was meticulously adjusted for optimal nulling of remote normal myocardium, late gadolinium enhancement imaging was completed.
